# Reappraisal of *ANK2* Variants in Cardiovascular Diseases: Uncovering Mechanisms and Future Directions

**DOI:** 10.31083/RCM26013

**Published:** 2025-01-15

**Authors:** Linjuan Guo, Dexi Wu, Wengen Zhu

**Affiliations:** ^1^Department of Cardiology, Jiangxi Provincial People's Hospital, The First Affiliated Hospital of Nanchang Medical College, 330006 Nanchang, Jiangxi, China; ^2^Department of Cardiology, The First Affiliated Hospital of Sun Yat-Sen University, 510080 Guangzhou, Guangdong, China

**Keywords:** *ANK2*, ankyrin-B, cardiac arrhythmias, cardiomyopathy, mechanism

## Abstract

Inherited cardiac arrhythmias, which may lead to sudden cardiac death, represent a significant health risk, with genetic factors playing a key role in their development. The ankyrin 2 (*ANK2*) gene, encoding ankyrin-B, is implicated in several heritable arrhythmia syndromes. *ANK2* variants have been linked to an inherited condition known as “ankyrin-B syndrome”, which manifests as a spectrum of cardiac arrhythmias and cardiomyopathy. Our current review examines the relationship between *ANK2* variants and specific heart conditions, summarizing recent findings on the genetic and molecular mechanisms underlying *ANK2*-related arrhythmias and structural abnormalities. By emphasizing the need for further research, this review aims to enhance understanding of *ANK2*’s role in heart disease and guide the development of effective therapies.

## 1. Introduction

Cardiac arrhythmias are common and potentially lead to sudden cardiac death (SCD) [1]. A previous study has linked genetic variants in ion channel genes to an increased risk of inheritable arrhythmia syndromes [2]. These genes are crucial for regulating the flow of electrically charged ions in and out of cells. Variants in these genes can affect the biophysical properties and trafficking of ion transporters and channels. A recent study has expanded the understanding of arrhythmias by focusing on genetic variants in non-ion channel genes [3]. One such gene is ankyrin 2 (*ANK2*), which is responsible for coding a protein known as ankyrin-B [4, 5, 6, 7].

Ankyrin-B, a member of the ankyrin protein family [7], is a protein that serves as an adapter essential for the proper expression and targeting of various integral membrane proteins in the heart, including cardiac ion channels, transporters, receptors, and signaling molecules [5]. The canonical structure of 220-kD ankyrin-B consists of several functional domains [8]. The N-terminal membrane-binding domain (MBD) comprises 24 ANK repeats, which are highly conserved and are involved in interactions between proteins. The spectrin-binding domain (SBD) is composed of a tandem of Zinc-binding domain 5 N-terminal-Zinc-binding domain 5 C-terminal-UPA (ZU5^N^-ZU5^C^-UPA) domains, facilitating interactions with a cytoskeletal protein called spectrin. The regulatory domain (RD) includes a death domain (DD) and a variable C-terminal domain (CTD) [8].

In humans, variants in the *ANK2* gene that result in the loss of function of ankyrin-B protein can significantly impact the activity of ion channels, leading to electrical instability in the heart. These variants have been linked to an inherited condition known as “ankyrin-B syndrome (ABS) [5, 9]”, which manifests as a spectrum of cardiac arrhythmias and can lead to SCD. ABS is inherited as an autosomal dominant trait and includes specific arrhythmias such as sinus node dysfunction, atrial fibrillation, long QT syndrome (LQTS) [10, 11], ventricular tachycardia (VT), idiopathic ventricular fibrillation (VF), catecholaminergic polymorphic ventricular tachycardia (CPVT), and SCD [5, 12, 13] (see **Supplementary Fig. 1**). Interestingly, recent studies have found that individuals carrying *ANK2* gene variants could have structural heart abnormalities [14, 15, 16, 17], regardless of whether they show a prolonged corrected QT (QTc) interval. This could potentially raise their risk of developing malignant arrhythmias and SCD.

Understanding the genetic basis of these conditions is essential for developing effective treatments and interventions for managing cardiac arrhythmias and SCD. Several studies [9, 11, 12] have investigated the role and mechanisms of *ANK2* gene variants in cardiovascular diseases. This review explores the relationship between *ANK2* gene variants and their associated cardiac phenotypes (Table 1), summarizes recent findings on genetic mechanisms and clinical impacts, and highlights areas for future research to enhance understanding and improve therapies for cardiovascular diseases.

**Table 1.** ***ANK2* Gene Variants Correlated with Cardiovascular Diseases in Recent Publications**.

Variants in *ANK2*	Location	Cardiac arrhythmias									Cardiac structure abnormalities				SCA/SCD	Family history	Functional analysis
		LQTS	CPVT	BrS	SND	WPW	CD	SVT/AF	VT/VF	TdP	DCM	HCM	ACM	CHD			
S646F	MBD	√				√					√			√		√	√
R990Q	SBD	√							√			√			√		√
Q1316H	SBD								√								√
T1437I	SBD	√						√		√							√
E1458G	SBD	√			√		√	√	√			√	√		√	√	√
M1988T	Exon 38												√		√		√
V3634D	RD	√	√	√	√			√	√		√				√	√	√
L3740I	RD	√	√						√							√	√
T3744N	RD	√						√					√			√	√
R3906W	RD	√			√			√		√		√				√	√
E3931K	RD	√				√	√	√	√	√		√			√	√	√
G53A	MBD										√				√		
S105T	MBD														√		
Y148H	MBD	√			√		√	√									
V248M	MBD					√		√				√					
G290S	MBD								√				√				
T466M	MBD								√				√				
G475R	MBD									√							
Q476R	MBD					√		√									
R539W	MBD										√					√	
V543M	MBD	√														√	
D683G	MBD										√			√			
V708M	MBD									√							
G761S	MBD	√			√											√	
L765M	MBD														√		
I777V	MBD	√															
T790I	MBD								√				√				
T825I	MBD			√													
G859R	MBD								√				√				
R895Q	MBD				√				√							√	
I964V	MBD							√					√				
R982Q	SBD			√													
L1128V	SBD			√	√		√									√	
R1305Q	SBD			√											√	√	
T1437M	SBD										√	√					
K1556*	SBD	√															
R1582Q	Exon 38												√				
V1593F	Exon 38														√		
R1604K	Exon 38	√															
K1626E	Exon 38			√													
H1806Q	Exon 38			√												√	
V1857E	Exon 38						√			√		√				√	
G1920R	Exon 38			√													
E1934V	Exon 38														√		
I2050T	Exon 38			√					√								
T2059M	Exon 38											√	√		√		
R2069H	Exon 38								√			√	√		√		
E2186K	Exon 38										√					√	
K2337E	Exon 38								√				√				
E2378K	Exon 38			√											√	√	
D2445G	Exon 38			√								√			√	√	
R2466H	Exon 38														√		
V2708A	Exon 38			√													
D2802H	Exon 38	√															
A2948G	Exon 38			√											√	√	
E3016K	Exon 38										√	√				√	
E3062G	Exon 38														√		
D3177H	Exon 38								√				√				
T3227P	Exon 38	√															
I3285T	Exon 38								√							√	
S3300R	Exon 38									√							
I3437T	Exon 38		√									√					
N3554Y	Exon 38								√				√				
E3570K	RD	√			√											√	
W3620R	RD	√		√	√				√	√						√	
L3621V	RD	√			√											√	
V3634I	RD	√														√	
E3650Q	RD											√			√		
R3659L	RD	√															
K3694R	RD												√				
E3696K	RD	√													√		
V3774M	RD			√													
T3776A	RD											√			√		
S3839T	RD	√															√
R3842W	RD											√			√		
I3940R	RD				√			√	√							√	

*ANK2*, ankyrin 2; LQTS, long QT syndrome; CPVT, catecholaminergic polymorphic ventricular tachycardia; BrS, Brugada syndrome; CD, conduction disorders; AF, atrial fibrillation; SVT, supraventricular tachycardia; WPW, Wolff Parkinson White Syndrome; SND, sinus node dysfunction; VT, ventricular tachycardia; VF, ventricular fibrillation; TdP, torsade de pointes; SCA, sudden cardiac arrest; SCD, sudden cardiac death; DCM, dilated cardiomyopathy; HCM, hypertrophic cardiomyopathy; ACM, arrhythmogenic cardiomyopathy; CHD, congenital heart disease; MBD, membrane-binding domain; SBD, spectrin-binding domain; RD, regulatory domain.

## 2. Functional Analysis for *ANK2* Variants

**Fig. 1.**
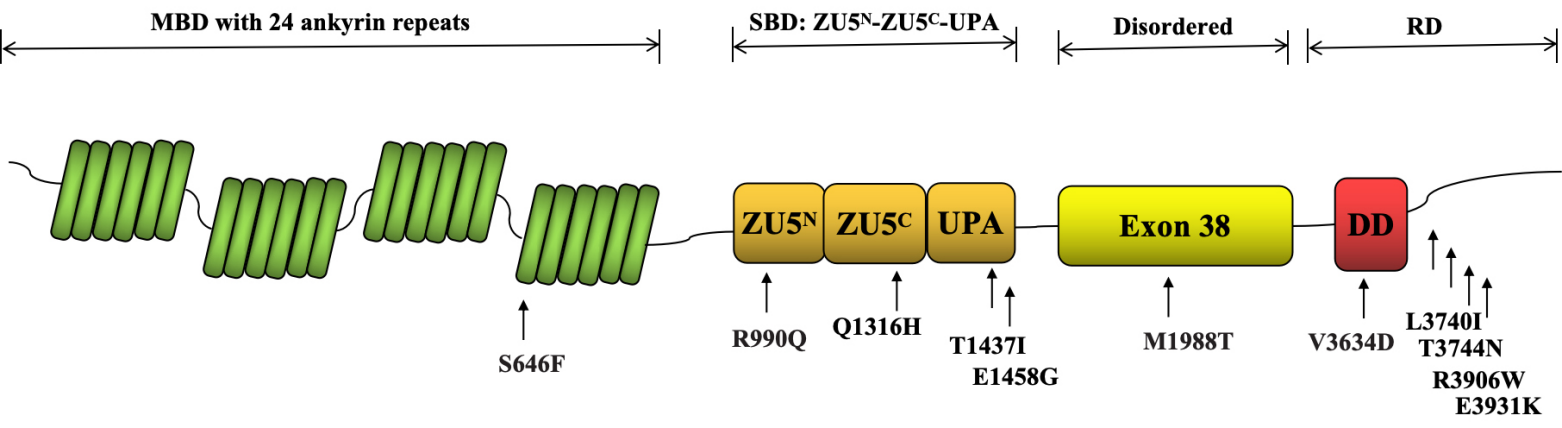
**Functional Analysis of *ANK2* Gene Variants Mapped to the NCBI Reference Sequence NP_001139.3**. The N-terminal MBD comprises 24 ankyrin repeats, which are highly conserved and are involved in interactions between proteins. The SBD is composed of a tandem of ZU5^N^-ZU5^C^-UPA domains. The disordered region (exon 38) is a flexible linker between the SBD and the RD. The RD includes a DD and a variable CTD. MBD, membrane-binding domain; SBD, spectrin-binding domain; RD, regulatory domain; CTD, C-terminal domain; DD, death domain; ZU5^N^-ZU5^C^-UPA, Zinc-binding domain 5 N-terminal-Zinc-binding domain 5 C-terminal-UPA; NCBI, National Center for Biotechnology Information.

Table 2 (Ref. [8]) presents the functional analysis of specific *ANK2* gene variants. To understand the roles and mechanisms of these *ANK2* variants, researchers employed a variety of methodologies and techniques. One approach evaluates the prevalence of the minor allele in different populations and examines co-segregation patterns to understand variant frequency and heritability. Protein sequence and biophysical modeling techniques are used to analyze ANK2 protein structure and predict how variants affect its function. *In silico* analyses further predict the effects of these variants on ankyrin-B. Furthermore, both *in vitro* and *in vivo* assays are crucial, with the latter involving the introduction of *ANK2* variants into mice to observe physiological responses and assess pathogenic potential. Despite the value of *in vivo* murine models, few studies have used them to analyze *ANK2* variants. Fig. 1 displays 11 *ANK2* gene variants, primarily located in the CTD, that have undergone functional analysis.

**Table 2.** **Functional Analysis of *ANK2* Gene Variants**.

*ANK2* gene variants			Annotation	dbSNP	Allele frequency (gnomAD)	Functional analysis				*ANK2* function		Downstream binding partners
*	^#^	※				*In silico* analysis	*In vitro* assays	Knock-out mice^&^	Knock-in mice	Expression	Localization	
S646F	S646F	S646F	Missense	rs786205724	-	√	√	×	×	Reduced	Abnormal	NCX1
R990Q	R990Q	R990Q	Missense	rs373261456	4.83251 × 10^-05^	√	√	√	×	NA	Abnormal	βII spectrin; NCX1
Q1316H	Q1316H	Q1283H	Missense	rs755373114	9.91229 × 10^-06^	√	√	×	√	Unchanged	Unchanged	PP2A/B56α-RyR2
T1437I	T1437I	T1404I	Missense	-	-	√	√	√	×	Unchanged	Unchanged	Na^+^/K^+^ ATPase; NCX1; IP3R
E1458G	E1458G	E1425G	Missense	rs72544141	7.4501 × 10^-04^	√	√	√	√	Reduced	Abnormal	Na^+^/K^+^ ATPase; NCX1; IP3R
M1988T	-	-	Missense	rs2154021231	6.20 × 10^-07^	√	√	×	×	Reduced	Abnormal	WNT/β-catenin pathway
V3634D	V1549D	V1516D	Missense	rs66785829	2.08879 × 10^-03^	√	√	√	×	Reduced	Abnormal	Na^+^/K^+^ ATPase; NCX1; IP3R
L3740I	L1655I	L1622I	Missense	rs35530544	1.753345 × 10^-03^	√	√	√	√	Unchanged	Unchanged	Na^+^/K^+^ ATPase; NCX1; IP3R
T3744N	T1659N	T1626N	Missense	rs121912705	1.171034 × 10^-03^	√	√	√	×	Unchanged	Unchanged	Na^+^/K^+^ ATPase; NCX1; IP3R
R3906W	R1821W	R1788W	Missense	rs121912706	1.250316 × 10^-03^	√	√	√	×	Reduced	Abnormal	Na^+^/K^+^ ATPase; NCX1; IP3R
E3931K	E1846K	E1813K	Missense	rs45454496	03.42896 × 10^-03^	√	√	√	×	Unchanged	Unchanged	Na^+^/K^+^ ATPase; NCX1; IP3R

*ANK2* variants were mapped to the following reference sequences: *NP_001139.3; ^#^NP_066187.2; and are associated with findings reported by ^※^Wang *et al*. [8] (DOI: 10.1073/pnas.1200613109). ^&^Introducing a mutant form of ankyrin-B into the myocytes of *ANK2* haploinsufficient mice does not restore normal function, indirectly supporting that these *ANK2* variants have a significant impact on the heart’s function. gnomAD, Genome Aggregation Database (http://gnomad.broadinstitute.org); NCX1, Na^+^/Ca^2+^ exchanger type 1; IP3R, inositol 1,4,5 trisphosphate receptor; PP2A, protein phosphatase 2A; RyR2, ryanodine receptor type 2; dbSNP, database of single nucleotide polymorphisms; NA, not available; WNT, wingless-type mouse mammary tumor virus integration site family; B56α-RyR2, protein phosphatase 2 regulatory subunit B56 alpha.

### 2.1 Pathogenic Mechanisms

In terms of the pathogenic mechanisms, the majority of *ANK2* variants have been found to disrupt the normal function of ankyrin-B in multiple ways. These variants affect the expression levels and cellular localization of ankyrin-B, which in turn disrupts the proper interaction with its downstream binding partners. The mechanisms behind reduced ankyrin-B expression remain unclear but are thought to involve autophagy and ubiquitin-proteasome degradation pathways [18, 19, 20].

A key partner affected by ankyrin-B dysfunction is the Na^+^/Ca^2+^ exchanger type 1 (NCX1) protein, essential for the regulation of intracellular calcium dynamics. Ankyrin-B dysfunction can cause erratic calcium dynamics, leading to cellular afterdepolarizations and extrasystoles that may trigger arrhythmias. In addition to NCX1, *ANK2* variants also affect other key binding partners, including the inositol 1,4,5-trisphosphate receptor (IP3R), Na^+^/K^+^ ATPase, protein phosphatase 2A (PP2A), and βII spectrin [5, 21, 22, 23], whose dysfunction exacerbates calcium imbalances (see **Supplementary Fig. 2**). As a result of the disrupted calcium dynamics, the affected individuals may experience cellular afterdepolarizations and extrasystoles [24, 25, 26]. These electrical disturbances in the cells can lead to arrhythmogenesis and abnormal heart rhythms.

However, the exact pathogenic mechanisms underlying *ANK2*-related cardiac structural abnormalities remain poorly understood. Further studies are needed to clarify how these variants contribute to structural abnormalities in the heart. This will enhance our understanding of ankyrin-B’s role in maintaining the heart’s structural and functional integrity.

### 2.2 Allele Frequency 

The frequency of a minor allele can serve as a valuable indicator in predicting a variant’s contribution to disease. If a variant is rare or absent in large population cohorts, it may indicate a potential disease-causing variant. Table 2 shows the allele frequencies of *ANK2* variants from the Genome Aggregation Database (gnomAD). Several *ANK2* variants previously reported in the literature show higher allele frequencies within the gnomAD population. The associations of some *ANK2* variants with diseases such as LQTS, Brugada Syndrome, and CPVT is debated over their pathogenicity, partly due to the high allele frequency of these *ANK2* variants in the general population [27, 28, 29, 30, 31].

Giudicessi and Ackerman [32] conducted a comprehensive evaluation of the genetic link between established loss-of-function *ANK2* variants and cardiac phenotypes in humans, which are often associated with ABS. They performed a retrospective review of medical records from 1727 patients referred to the Mayo Clinic Genetic Heart Rhythm Clinic to identify individuals carrying alleged ABS-causative *ANK2* variants. These variants were mapped to the current reference transcript (NP_001139.3) and their frequencies compared with gnomAD data. Among 1727 patients, only 12 (0.7%) carried 4 *ANK2* variants (p.E1458G, p.V3634D, p.T3744N, p.R3906W) linked to ABS. These variants were not significantly enriched in clinical settings and showed higher frequencies in public exomes/genomes reported by gnomAD. Further examination revealed that only a few of the 12 cases experienced cardiac events possibly related to ABS, while most remained asymptomatic. This suggests that these *ANK2* variants are not likely to cause a monogenic condition predisposing an individual to SCD. Due to limited access to detailed genetic ancestry and clinical data, further genotype-phenotype studies—potentially through a multicenter registry—are needed to clarify the role of *ANK2* loss-of-function in ABS.

### 2.3 In Vivo Functional Analysis

Minor allele frequency (MAF) cut-offs, such as 1% in the general population, are often used to suggest potential pathogenicity, with lower frequencies indicating rarer and possibly more pathogenic variants. In addition, the threshold can be as high as 5% in studies considering population-specific effects. However, MAF is not a definitive measure, as gene variants with high MAF (e.g., *ANK2* gene) can still be disease-associated under certain conditions. Therefore, beyond MAF, assessing pathogenicity should consider other supporting evidence such as family history and functional data.

Heterozygous *ANK2* knockout murine models [9, 11], exhibiting *ANK2* haploinsufficiency show dysregulated calcium levels, resulting in various complex cardiac phenotypes. These phenotypes include atrial arrhythmias, sinus bradycardia, QTc interval prolongation, catecholamine-induced ventricular arrhythmias, and an elevated risk of SCD. Similar phenotypes are observed in patients with ABS associated with *ANK2* variants. The introduction of a mutant ankyrin-B into the myocytes of *ANK2* haploinsufficient mice fails to restore normal cardiac function, suggesting the pathogenicity of certain *ANK2* variants.

Most *in vivo* functional analyses of *ANK2* variants, as shown in Table 2, currently rely on *ANK2* haploinsufficient mice. However, these models may not fully reflect the disease risks associated with *ANK2* variants in humans. Therefore, the direct effects of *ANK2* variants on cardiac structure and function remain unclear and warrant further investigation. Knock-in mouse models carrying specific *ANK2* variants can provide deeper insights into their influence on cardiac structure and function. These models more closely replicate the genetic alterations seen in humans, aiding in accurately defining associated disease risks and advancing the understanding of the pathophysiological mechanisms of *ANK2* variants. To date, only three *ANK2* variants—p.L3740I [33], p.E1458G [34], and p.Q1316H [35]—have been engineered into knock-in mouse models for functional analysis. As a result, the *in vivo* effects and arrhythmogenic potential of *ANK2* variants remain largely unexplored, necessitating further investigation.

A previous study suggests that murine models may exaggerate the physiological impact of *ANK2* loss-of-function variants observed in humans [32]. Given the species-specific differences between humans and animals, there is a need for future research using models that better reflect the human condition, particularly those incorporating disease-causing *ANK2* variants. This includes using patient-derived induced pluripotent stem cells, which could provide a more accurate representation of human genetic alterations and help define associated disease risks.

## 3. *ANK2* Variants in Patients with Cardiovascular Diseases

Table 1 provides a comprehensive list of known *ANK2* gene variants associated with cardiovascular diseases, as identified in the previously published studies (see **Supplementary references**). These specific variants have been mapped to the NCBI reference transcript: NP_001139.3. These *ANK2* gene variants are associated with a range of cardiac arrhythmias and structural cardiac abnormalities. The distribution of these variants can be observed across different protein domains, specifically the MBD, SBD, and CTD.

### 3.1 ANK2 Variants and Cardiac Arrhythmias

Prolonged QTc intervals, whether congenital or acquired LQTS, are frequently identified in patients with *ANK2* variants. The p.E1458G variant of *ANK2*, previously denoted as p.E1425G [11], is among the earliest variant linked to prolonged QTc intervals and was initially classified as LQTS type 4. This specific variant is found to segregate with the long-QT phenotype in 22 out of 24 individuals in the studied family, thereby indicating a significant genetic link to LQTS. Functionally, the p.E1458G variant disrupts the normal function of ankyrin-B, altering calcium signaling in cardiomyocytes and potentially precipitating extrasystoles, which may explain the arrhythmias in affected individuals. This research highlights ankyrin-B as the first non-ion channel protein implicated in inherited arrhythmias. The discovery of ankyrin-B’s role in LQTS introduces a new pathophysiological mechanism and broadens our understanding of inherited arrhythmias, with implications for targeted therapy development.

While debates continue on how *ANK2* variants influence the QTc interval, it is important to note that not all individuals with *ANK2* variants experience a prolonged QTc interval. Indeed, loss-of-function variants in the *ANK2* gene are linked to various cardiac arrhythmias beyond LQTS. These include sinus node dysfunction [9, 11, 36, 37, 38], conduction disorders [13, 39], atrial fibrillation [9, 40], ventricular tachycardia/ventricular fibrillation, CPVT [13, 36], Wolff-Parkinson-White Syndrome (WPW) [41], Brugada Syndrome [42], and Torsade de Pointes [9, 43] (Table 1).

The mechanisms underlying the diversity of arrhythmia phenotypes associated with *ANK2* variants are not fully understood and remain a focus of ongoing research. The clinical variability associated with *ANK2* mutations may partly arise from complex interactions between ankyrin-B and other critical cellular binding partners essential for maintaining cellular structure and function. Notably, a single *ANK2* variant can result in different arrhythmia phenotypes among affected individuals, a common occurrence in genetic diseases. The same variant does not uniformly manifest in all individuals due to varying penetrance and expressivity, influenced by modifying genes.

Indeed, *ANK2* variants are often found alongside other genetic mutations in an individual’s genome [14, 15, 44]. For example, the *ANK2* p.E3931K variant has been shown to exacerbate the cardiac phenotype in individuals carrying the *KCNH2* p.H562R variant [44]. In isolation, the p.E3931K variant is linked to age-related conduction diseases, while individuals with only the *KCNH2* p.H562R variant typically show no symptoms. This suggests that the cumulative effects of multiple genetic variants—*ANK2* and *KCNH2* in this case—could influence the manifestation and severity of the disease. This raises the possibility that *ANK2* variants may play a key role in a complex disease etiology that could be oligogenic or polygenic. Given the complexity involved, further research is essential to understand how *ANK2* variants contribute to the pathophysiology of arrhythmias.

### 3.2 ANK2 Variants and Cardiac Structure Abnormalities

Beyond various cardiac arrhythmias, loss-of-function variants in the *ANK2* gene are linked to structural abnormalities, such as hypertrophic cardiomyopathy (HCM) [14], dilated cardiomyopathy (DCM) [17], arrhythmogenic cardiomyopathy (ACM) [45], and congenital heart disease [16]. A prior study conducted by Lopes *et al*. [14] investigated the genotype-phenotype correlations in HCM by employing high-throughput sequencing to analyze genetic variants. This study involved a thorough clinical assessment and genetic examination of 41 inherited heart disease-associated genes across 874 unrelated and consecutively recruited patients. The findings revealed a significant link between rare *ANK2* variants and the severity of left ventricular hypertrophy in HCM. Specifically, carriers of these *ANK2* variants exhibited significantly greater maximum left ventricular wall thickness. These findings suggest the potential role of *ANK2* in modulating the clinical expression of the HCM phenotype. Although direct causality between *ANK2* gene variants and alterations in left ventricular morphology is not established, *ANK2* may exert an indirect effect on cellular phenotype through interactions with proteins involved in calcium regulation and β-adrenergic signaling. While further validation in separate cohorts is required to confirm these findings, Lopes *et al*. [14] provide a foundational understanding of *ANK2*’s role in HCM.

Roberts *et al*. [15] reported a case of arrhythmogenic right ventricular cardiomyopathy (ARVC) found during an autopsy of an individual with ABS. Further genetic screening through exome sequencing identified a novel *ANK2* variant (p.M1988T) in the ARVC-affected family. Subsequently, a comprehensive genetic screen identified several rare *ANK2* variants in a cohort of patients with genotype-negative ARVC, indicating the presence of the disease phenotype without known pathogenic variants in conventional ARVC-associated genes. Probands with these rare *ANK2* variants exhibit ventricular arrhythmias and structural abnormalities such as biventricular dilation and cardiac fibrosis. Functionally, while the direct functions and mechanisms of the *ANK2* variants were not explored, the researchers observed severe cardiac abnormalities characteristic of ACM in the *ANK2*-cKO mouse model, a genetically engineered model with cardiomyocyte-specific deletion of ankyrin-B. These abnormalities primarily included biventricular dilation, reduced ejection fraction, cardiac fibrosis, premature death, sinus bradycardia, QTc interval prolongation, and catecholamine-induced ventricular arrhythmias. Interestingly, despite the absence of fatty infiltration typically seen in human ACM, *ANK2*-cKO mice exhibited a preserved desmosomal structure, suggesting that ankyrin-B dysfunction may trigger ACM through a distinct pathophysiological mechanism. This study further emphasized the disrupted interaction between ankyrin-B and β-catenin in *ANK2*-cKO mice. Therapeutically, treatment with SB-216763, an activator of the wingless-type mouse mammary tumor virus integration site family (WNT)/β-catenin pathway, prevented and partially reversed ACM phenotypes in these mice. Collectively, these findings underscore the importance of ankyrin-B in ACM genesis and pave the way for developing targeted therapeutics. However, the exact roles and mechanisms by which *ANK2* variants contribute to ACM pathogenesis remain to be fully elucidated.

Swayne and colleagues [16] conducted a comprehensive study on the *ANK2* p.S646F variant, which was identified in two multigenerational families from the Gitxsan First Nation, a community with a high incidence of LQTS. This variant, located within the MBD of the *ANK2* gene, is associated with a spectrum of cardiac phenotypes, including LQTS, WPW, congenital heart malformations, and DCM. The p.S646F variant leads to loss of ankyrin-B function, resulting in decreased expression levels and abnormal cellular localization in both cultured H9c2 cells and primary cardiomyocytes. The expression levels of ankyrin-B might be modulated by the proteasome and the p.S646F variant could impair cell viability [18]. Furthermore, *ANK2* p.S646F adversely affects the proper membrane targeting of the NCX1, a key binding partner of ankyrin-B, indicating disrupted interaction between ankyrin-B and its downstream targets. This research identifies *ANK2* p.S646F as a significant genetic factor in various cardiac phenotypes, emphasizing the essential role of ankyrin-B in cardiac structure and function.

While these studies illuminate the potential influence of *ANK2* variants, their exact impact on structural heart diseases remains largely uncertain [17], emphasizing the urgent need for further exploration. Currently, many of these variants are classified as variants of uncertain significance, highlighting the need for ongoing research. Future studies are crucial to delineate the specific contributions of these *ANK2* variants for the onset and progression of structural heart diseases, offering critical insights that could inform therapeutic strategies.

## 4. Future Directions

As gene sequencing technology continues to advance, a growing number of *ANK2* variants, including both coding and non-coding types, are expected to be identified. While most reported variants are heterozygous missense mutations, attention should also be directed toward other mutation types, such as homozygous mutations. Further investigation is warranted to elucidate the roles of these *ANK2* variants in cardiovascular diseases, particularly regarding their influence on the development and progression of structural heart diseases [15, 16]. Given that many *ANK2* variants are currently classified as variants of uncertain significance, it is crucial to enhance our understanding of the pathophysiological mechanisms linking these variants to cardiac abnormalities and arrhythmias.

Although *in vivo* murine models have provided valuable insights, their ability to accurately represent the disease risks associated with *ANK2* variants in humans may be limited. There is also evidence suggesting that these models may exaggerate the physiological impact of *ANK2* loss-of-function variants in humans [32]. This underscores the necessity for research that considers species-specific differences and the development of more human-relevant models. Patient-derived induced pluripotent stem cells could better reflect human genetic alterations, facilitating the precise determination of disease risks. Future studies should also explore the potential for *ANK2* variants, in combination with other genetic factors, to contribute to a complex disease etiology that could be oligogenic or polygenic, broadening our understanding of cardiovascular disease genetics. Additionally, further genotype-phenotype association studies, potentially involving multicenter registries, are essential to clarify the role of loss-of-function *ANK2* variants in ABS pathogenesis.

Both canonical and noncanonical (giant) ankyrin-G variants have been identified as crucial for cardiovascular function and overall heart health [46, 47]. Canonical ankyrin-B is essential for normal cardiac function, while noncanonical giant ankyrin-B isoforms are linked to neurological disorders [48, 49, 50]. Despite this, the specific role of giant ankyrin-B in cardiovascular tissues remains largely unexplored. However, the specific role of giant ankyrin-B in cardiovascular tissues remains largely unexplored. Preliminary findings have identified certain variants within the giant *ANK2* gene, particularly in exon 38 (e.g., *ANK2* p.M1988T), among patients with cardiovascular diseases. However, the precise role and underlying mechanisms of these variants remain to be elucidated. The precise role and underlying mechanisms of these variants require further investigation to elucidate the function of giant ankyrin-B in cardiovascular diseases.

According to the Human Protein Atlas (https://www.proteinatlas.org), the *ANK2* gene is most highly expressed in cardiomyocytes, followed by fibroblasts, endothelial cells, macrophages, and T-cells. While cardiomyocytes have been the focus of *ANK2* research, the potential role of *ANK2* in other myocardial cells in heart diseases is an area that requires further investigation.

## 5. Conclusions

Despite progress in identifying *ANK2* variants and their potential implications in cardiovascular diseases, significant gaps in our understanding persist. Future research should focus on elucidating the precise mechanisms by which *ANK2* variants contribute to disease. This should involve models that closely mimic human biology and examine the cumulative effects of multiple genetic variants on disease presentation and severity.

## Supplementary Material

Supplementary material associated with this article can be found, in the online version, at https://doi.org/10.31083/RCM26013.
