# Supplementary material for: Reappraisal of ANK2 Variants in Cardiovascular Diseases: Uncovering Mechanisms and Future Directions
Source: Rev Cardiovasc Med. 2025 Jan 15;26(1):26013. doi: 10.31083/RCM26013 (PMC11759970; doi:10.31083/RCM26013)
Supplement: Supplementary file 1 [file 2153-8174-26-1-26013-s1.doc]

**Supplementary references: Overview of Published Studies on ANK2 Gene Variants Linked to Cardiovascular Diseases**

1. Mohler PJ, Schott JJ, Gramolini AO, Dilly KW, Guatimosim S, DuBell WH, Song LS, Haurogne K, Kyndt F, Ali ME, Rogers TB, Lederer WJ, Escande D, Le Marec H, Bennett V. Ankyrin-B mutation causes type 4 long-QT cardiac arrhythmia and sudden cardiac death. NATURE. 2003;421(6923):634-639.

2. Mohler PJ, Splawski I, Napolitano C, Bottelli G, Sharpe L, Timothy K, Priori SG, Keating MT, Bennett V. A cardiac arrhythmia syndrome caused by loss of ankyrin-B function. Proc Natl Acad Sci U S A. 2004;101(24):9137-9142.

3. Sherman J, Tester DJ, Ackerman MJ. Targeted mutational analysis of ankyrin-B in 541 consecutive, unrelated patients referred for long QT syndrome genetic testing and 200 healthy subjects. HEART RHYTHM. 2005;2(11):1218-1223.

4. Zhou X, Shimizu M, Konno T, Ino H, Fujino N, Uchiyama K, Mabuchi T, Kaneda T, Fujita T, Masuda E, Kato H, Funada A, Mabuchi H. Analysis of ankyrin-B gene mutations in patients with long QT syndrome. Nan Fang Yi Ke Da Xue Xue Bao. 2006;26(7):901-903, 909.

5. Mohler PJ, Le Scouarnec S, Denjoy I, Lowe JS, Guicheney P, Caron L, Driskell IM, Schott J, Norris K, Leenhardt A, Kim RB, Escande D, Roden DM. Defining the Cellular Phenotype of “Ankyrin-B Syndrome” Variants. CIRCULATION. 2007;115(4):432-441.

6. Zhu W, Wang C, Hu J, Wan R, Yu J, Xie J, Ma J, Guo L, Ge J, Qiu Y, Chen L, Liu H, Yan X, Liu X, Ye J, He W, Shen Y, Wang C, Mohler PJ, Hong K. Ankyrin-B Q1283H Variant Linked to Arrhythmias Via Loss of Local Protein Phosphatase 2A Activity Causes Ryanodine Receptor Hyperphosphorylation. CIRCULATION. 2018;138(23):2682-2697.

7. Shigemizu D, Aiba T, Nakagawa H, Ozaki K, Miya F, Satake W, Toda T, Miyamoto Y, Fujimoto A, Suzuki Y, Kubo M, Tsunoda T, Shimizu W, Tanaka T. Exome Analyses of Long QT Syndrome Reveal Candidate Pathogenic Mutations in Calmodulin-Interacting Genes. PLOS ONE. 2015;10(7):e130329.

8. Sanchez O, Campuzano O, Fernández-Falgueras A, Sarquella-Brugada G, Cesar S, Mademont I, Mates J, Pérez-Serra A, Coll M, Pico F, Iglesias A, Tirón C, Allegue C, Carro E, Gallego MÁ, Ferrer-Costa C, Hospital A, Bardalet N, Borondo JC, Vingut A, Arbelo E, Brugada J, Castellà J, Medallo J, Brugada R. Natural and Undetermined Sudden Death: Value of Post-Mortem Genetic Investigation. PLOS ONE. 2016;11(12):e167358.

9. Saprungruang A, Khongphatthanayothin A, Mauleekoonphairoj J, Wandee P, Kanjanauthai S, Bhuiyan ZA, Wilde AAM, Poovorawan Y. Genotype and clinical characteristics of congenital long QT syndrome in Thailand. Indian Pacing and Electrophysiology Journal. 2018.

10. Giudicessi JR, Ackerman MJ. Established Loss-of-Function Variants inANK2 -Encoded Ankyrin-B Rarely Cause a Concerning Cardiac Phenotype in Humans. Circulation: Genomic and Precision Medicine. 2020;13(2).

11. Coban Akdemir ZH, Charng WL, Azamian M, Paine IS, Punetha J, Grochowski CM, Gambin T, Valdes SO, Cannon B, Zapata G, Hernandez PP, Jhangiani S, Doddapaneni H, Hu J, Boricha F, Muzny DM, Boerwinkle E, Yang Y, Gibbs RA, Posey JE, Wehrens XHT, Belmont JW, Kim JJ, Miyake CY, Lupski JR, Lalani SR. Wolff–Parkinson–White syndrome: De novo variants and evidence for mutational burden in genes associated with atrial fibrillation. American journal of medical genetics. Part A. 2020;182(6):1387-1399.

12. Song J, Sasmita BR, Deng G. Ankyrin-2 genetic variants: A case of Ankyrin-B syndrome. Ann Noninvasive Electrocardiol. 2022;27(4):e12933.

13. von Korn H, Basso C, Pilichou K, Stefan V, Swojanowsky P. A New Inherited Syndrome Causing Sudden Cardiac Death with Distinct ST-Segment Depression and Ankyrin-2-Mutation. Appl Clin Genet. 2023;16:233-239.

14. Lieve KV, Williams L, Daly A, Richard G, Bale S, Macaya D, Chung WK. Results of Genetic Testing in 855 Consecutive Unrelated Patients Referred for Long QT Syndrome in a Clinical Laboratory. GENET TEST MOL BIOMA. 2013;17(7):553-561.

15. Freudenberg-Hua Y, Freudenberg J, Vacic V, Abhyankar A, Emde AK, Ben-Avraham D, Barzilai N, Oschwald D, Christen E, Koppel J, Greenwald B, Darnell RB, Germer S, Atzmon G, Davies P. Disease variants in genomes of 44 centenarians. Mol Genet Genomic Med. 2014;2(5):438-450.

16. Krogh Broendberg A, Pedersen LN, Nielsen JC, Jensen HK. Ankyrin-2 variants associated with idiopathic ventricular fibrillation storm in patients with intermittent early repolarization pattern. HeartRhythm Case Reports. 2015;1(5):337-341.

17. Allegue C, Coll M, Mates J, Campuzano O, Iglesias A, Sobrino B, Brion M, Amigo J, Carracedo A, Brugada P, Brugada J, Brugada R. Genetic Analysis of Arrhythmogenic Diseases in the Era of NGS: The Complexity of Clinical Decision-Making in Brugada Syndrome. PLOS ONE. 2015;10(7):e133037.

18. Farrugia A, Keyser C, Hollard C, Raul JS, Muller J, Ludes B. Targeted next generation sequencing application in cardiac channelopathies: Analysis of a cohort of autopsy-negative sudden unexplained deaths. FORENSIC SCI INT. 2015;254:5-11.

19. Ichikawa M, Aiba T, Ohno S, Shigemizu D, Ozawa J, Sonoda K, Fukuyama M, Itoh H, Miyamoto Y, Tsunoda T, Makiyama T, Tanaka T, Shimizu W, Horie M. Phenotypic Variability of ANK2 Mutations in Patients With Inherited Primary Arrhythmia Syndromes. CIRC J. 2016;80(12):2435-2442.

20. Watanabe H, Minamino T. Rare Variants in ANK2 Associated With Various Inherited Arrhythmia Syndromes. CIRC J. 2016;80(12):2423-2424.

21. Maltese PE, Orlova N, Krasikova E, Emelyanchik E, Cheremisina A, Kuscaeva A, Salmina A, Miotto R, Bonizzato A, Guerri G, Zuntini M, Nicoulina S, Bertelli M. Gene-Targeted Analysis of Clinically Diagnosed Long QT Russian Families. INT HEART J. 2016.

22. Hata Y, Kinoshita K, Mizumaki K, Yamaguchi Y, Hirono K, Ichida F, Takasaki A, Mori H, Nishida N. Postmortem genetic analysis of sudden unexplained death syndrome under 50 years of age: A next-generation sequencing study. HEART RHYTHM. 2016;13(7):1544-1551.

23. Asadi M, Foo R, Samienasab M, Salehi A, Kheirollahi M, Khanahmad H, Salehi R. Genetic analysis of Iranian family with hereditary cardiac arrhythmias by next generation sequencing. Advanced Biomedical Research. 2016;5(1):55.

24. Hata Y, Yoshida K, Kinoshita K, Nishida N. Epilepsy-related sudden unexpected death: targeted molecular analysis of inherited heart disease genes using next-generation DNA sequencing. BRAIN PATHOL. 2017;27(3):292-304.

25. Nishiyama T, Aizawa Y, Ito S, Katsumata Y, Kimura T, Takatsuki S. A subtype of idiopathic ventricular fibrillation and its relevance to catheter ablation and genetic variants. HeartRhythm Case Reports. 2017;3(5):277-281.

26. Campuzano O, Sanchez-Molero O, Mademont-Soler I, Coll M, Allegue C, Ferrer-Costa C, Mates J, Perez-Serra A, Del Olmo B, Iglesias A, Sarquella-Brugada G, Brugada J, Borondo JC, Castella J, Medallo J, Brugada R. Genetic analysis in post-mortem samples with micro-ischemic alterations. FORENSIC SCI INT. 2017;271:120-125.

27. Gessner G, Runge S, Koenen M, Heinemann SH, Koenen M, Haas J, Meder B, Thomas D, Katus HA, Schweizer PA. ANK2 functionally interacts with KCNH2 aggravating long QT syndrome in a double mutation carrier. BIOCHEM BIOPH RES CO. 2019;512(4):845-851.

28. Marketou ME, Zareas I, Kanoupakis E, Patrianakos A, Parthenakis F. A case series of Brugada syndrome with a novel mutation in the ankyrin-B gene: an unusual unmasking in acute myocarditis. European Heart Journal - Case Reports. 2021;5(6).

29. Bora E, Bulut AY, Cankaya T, Cinleti T, Genc HZ, Ozcan EE, Ozpelit E, Ulgenalp A, Caglayan AO. Clinical Heterogeneity in Patients with Long QT Syndrome and Segregation of Single Nucleotide Variants and Clinical Symptoms in 17 Affected Families. Mol Syndromol. 2023;14(5):363-374.

30. Tse G, Lee S, Zhou J, Liu T, Wong ICK, Mak C, Mok NS, Jeevaratnam K, Zhang Q, Cheng SH, Wong WT. Territory-Wide Chinese Cohort of Long QT Syndrome: Random Survival Forest and Cox Analyses. Frontiers in Cardiovascular Medicine. 2021;8.

31. Gama M, Cardoso I, Palma AM, Aguiar RS, Gaspar DCP, Fortuna P. Arrhythmogenic Left Ventricular Cardiomyopathy: A Successful Case of Extracorporeal Cardiopulmonary Resuscitation. Acta Med Port. 2023;36(9):598-602.

32. Mank-Seymour AR, Richmond JL, Wood LS, Reynolds JM, Fan YT, Warnes GR, Milos PM, Thompson JF. Association of torsades de pointes with novel and known single nucleotide polymorphisms in long QT syndrome genes. AM HEART J. 2006;152(6):1116-1122.

33. Lopes LR, Syrris P, Guttmann OP, O'Mahony C, Tang HC, Dalageorgou C, Jenkins S, Hubank M, Monserrat L, McKenna WJ, Plagnol V, Elliott PM. Novel genotype–phenotype associations demonstrated by high-throughput sequencing in patients with hypertrophic cardiomyopathy. HEART. 2015;101(4):294-301.

34. Swayne LA, Murphy NP, Asuri S, Chen L, Xu X, McIntosh S, Wang C, Lancione PJ, Roberts JD, Kerr C, Sanatani S, Sherwin E, Kline CF, Zhang M, Mohler PJ, Arbour LT. Novel Variant in theANK2 Membrane-Binding Domain Is Associated With Ankyrin-B Syndrome and Structural Heart Disease in a First Nations Population With a High Rate of Long QT Syndrome. Circulation: Cardiovascular Genetics. 2017;10(1):e1537.

35. Forleo C, D Erchia AM, Sorrentino S, Manzari C, Chiara M, Iacoviello M, Guaricci AI, De Santis D, Musci RL, La Spada A, Marangelli V, Pesole G, Favale S. Targeted next-generation sequencing detects novel gene–phenotype associations and expands the mutational spectrum in cardiomyopathies. PLOS ONE. 2017;12(7):e181842.

36. Roberts JD, Murphy NP, Hamilton RM, Lubbers ER, James CA, Kline CF, Gollob MH, Krahn AD, Sturm AC, Musa H, El-Refaey M, Koenig S, Aneq MÅ, Hoorntje ET, Graw SL, Davies RW, Rafiq MA, Koopmann TT, Aafaqi S, Fatah M, Chiasson DA, Taylor MRG, Simmons SL, Han M, van Opbergen CJM, Wold LE, Sinagra G, Mittal K, Tichnell C, Murray B, Codima A, Nazer B, Nguyen DT, Marcus FI, Sobriera N, Lodder EM, van den Berg MP, Spears DA, Robinson JF, Ursell PC, Green AK, Skanes AC, Tang AS, Gardner MJ, Hegele RA, van Veen TAB, Wilde AAM, Healey JS, Janssen PML, Mestroni L, van Tintelen JP, Calkins H, Judge DP, Hund TJ, Scheinman MM, Mohler PJ. Ankyrin-B dysfunction predisposes to arrhythmogenic cardiomyopathy and is amenable to therapy. J CLIN INVEST. 2019;129(8):3171-3184.

37. Sedlacek K, Stark K, Cunha SR, Pfeufer A, Weber S, Berger I, Perz S, Kaab S, Wichmann HE, Mohler PJ, Hengstenberg C, Jeron A. Common genetic variants in ANK2 modulate QT interval: results from the KORA study. Circ Cardiovasc Genet. 2008;1(2):93-99.


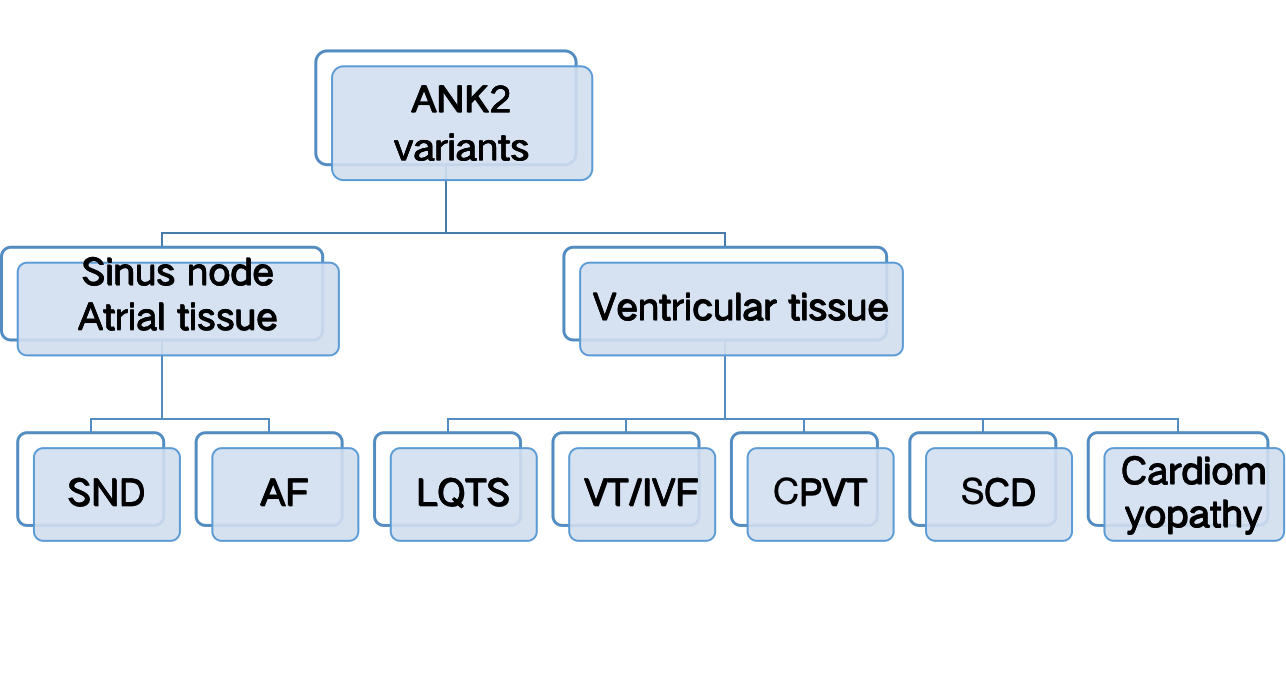


**Supplementary Fig. 1.** ANK2 variants have been linked to a inherited condition known as "ankyrin-B syndrome," which manifests as a spectrum of cardiac arrhythmias and cardiomyopathy.

SND: Sinoatrial Node Dysfunction

AF: Atrial Fibrillation

LQTS4: Long QT Syndrome type 4

VT: Ventricular Tachycardia

IVF: Idiopathic Ventricular Fibrillation

CPVT: Catecholaminergic Polymorphic Ventricular Tachycardia

SCD: Sudden Cardiac Death


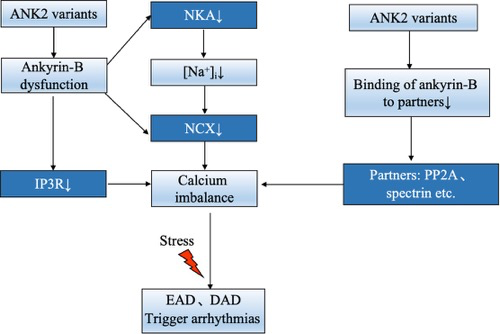


**Supplementary Fig. 2.** Pathogenic mechanisms of ANK2 variants in the development of arrhythmias.

ANK2: Ankyrin-2

NKA: Sodium-Potassium ATPase (Na⁺/K⁺-ATPase)

NCX: Sodium-Calcium Exchanger

IP3R: Inositol 1,4,5-trisphosphate receptor

PP2A: Protein Phosphatase 2A

EAD: Early Afterdepolarizations

DAD: Delayed Afterdepolarizations
